# Impact of ventricular pacing modalities and pacing percentage on new-onset atrial fibrillation after dual-chamber pacemaker implantation

**DOI:** 10.3389/fcvm.2025.1615420

**Published:** 2025-06-19

**Authors:** Meng Zhang, Jian Xu, Jianhua Wang, Yuxin Liu, Yuyang Jin, Shufeng Li, Wei Cao

**Affiliations:** Department of Cardiology, The Second Affiliated Hospital of Harbin Medical University, Harbin, China

**Keywords:** atrial fibrillation, left bundle branch pacing, right ventricular septal pacing, ventricular pacing percentage, dual-chamber pacemaker implantation

## Abstract

**Objective:**

To investigate the impact of the ventricular pacing modalities and ventricular pacing percentage (VP) on the incidence of new-onset atrial fibrillation (AF) following dual-chamber pacemaker implantation. We aim to explore how these factors, along with left atrial diameter (LAD), contribute to AF development, providing insights into optimizing pacemaker settings to potentially reduce AF risk in pacemaker patients.

**Methods:**

Between January 1, 2020, and December 31, 2022, a total of 371 patients who underwent initial dual-chamber pacemaker implantation at the Second Affiliated Hospital of Harbin Medical University were enrolled. These patients were categorized into two groups: the non-AF group and the AF group, based on the presence of new-onset AF during one-year follow-up. Regular remote follow-up visits were conducted postoperatively, with endpoint events defined as the detection of AF. A comparative analysis was performed to evaluate the differences in basic characteristics, implantation site of the ventricular lead, and pacemaker programming settings between the two groups.

**Results:**

The AF group exhibited a significantly higher prevalence of hypertension and a greater proportion of left bundle branch pacing (LBBP) compared to the non-AF group. Additionally, the AF group had a lower proportion of patients with ventricular pacing (VP) in the 0%–39% range, but a higher proportion of patients in the 40%–79% range. Notably, the LBBP cohort demonstrated a significantly lower incidence of AF. The AF group also presented with a larger left atrial diameter (LAD). Multivariate analysis revealed that LBBP and VP in the 0%–39% range were independently associated with a reduced risk of new-onset atrial fibrillation AF. Among the predictive indicators for new-onset AF, both LBBP and LAD demonstrated notable sensitivity and specificity.

**Conclusions:**

Ventricular pacing modalities and percentage significantly impact new-onset AF after dual-chamber pacemaker implantation. LBBP and lower VP percentages are associated with a lower risk of AF development, whereas a larger LAD may be linked to an increased likelihood of its onset. Optimizing these factors could potentially reduce the risk of AF in pacemaker patients.

## Introduction

Sick sinus syndrome (SSS) and atrioventricular block (AVB) are leading indications for pacemaker implantation globally (1). For patients with SSS and AVB requiring permanent pacing, dual-chamber pacing is generally preferred over single-chamber ventricular pacing (2). A comprehensive Cochrane review evaluating pacing modes and patient outcomes concluded that dual-chamber pacing is favored due to its lower incidence of atrial fibrillation (AF) and the reduced occurrence of pacemaker syndrome compared to single-chamber ventricular pacing (2).

Despite this, AF remains the most common cardiac arrhythmia in clinical practice. AF not only leads to a high volume of healthcare consultations but also significantly increases patient morbidity and mortality. The widespread prevalence and serious consequences of AF highlight its critical role as a major public health challenge in modern cardiology (3). The mechanisms by which a dual-chamber pacemaker may induce AF are complex and not yet fully understood. These mechanisms include mechanical stimulation by the pacing electrode such as the ventricular pacing percentage (VP) (4), pacing in the right atrium leading to delayed activation of the left atrium (5), pacemaker-mediated tachycardia that may vary with different ventricular pacing modalities (6), and long-term atrial pacing potentially causing local inflammation or fibrosis (7). The overall pathophysiological processes involved are intricate and still require further clarification.

This study aims to investigate the impact of the ventricular pacing modalities and VP on the incidence of new-onset AF following dual-chamber pacemaker implantation.

## Methods

### Ethics statement

This study protocol conforms to the ethical guidelines of the 1975 Declaration of Helsinki as reflected in *a priori* approval by the institution's human research committee. was approved by the Research Ethics Committee of the Second Affiliated Hospital of Harbin Medical University. Informed consent was obtained from each patient.

### Study population

A retrospective analysis was conducted on 870 patients who underwent initial dual-chamber pacemaker implantation at Department of Cardiology, the Second Affiliated Hospital of Harbin Medical University from January 1, 2020, to December 31, 2022. Of these, 371 patients met the inclusion criteria and were enrolled in the study. Participants were categorized into two groups: the AF group and the non-AF group. In this study, AF is defined as clinical AF. The diagnosis of clinical AF requires rhythm documentation via an electrocardiogram (ECG) showing characteristic AF patterns. According to established guidelines, an episode lasting at least 30 s is considered diagnostic of clinical AF. A standard 12-lead ECG or a single-lead ECG tracing of more than 30 s, demonstrating an irregular heart rhythm with no discernible, repetitive P waves and irregular RR intervals (provided atrioventricular conduction is not impaired), is diagnostic for clinical AF (8).

Inclusion Criteria: (i) patients receiving dual-chamber pacemaker implantation; (ii) patients meeting Class I or IIa indications for pacemaker implantation due to conditions such as SSS or AVB (2); (iii) age 18 years or older; (iv) willingness and ability to participate in the study. Exclusion Criteria: (i) preoperative history of atrial fibrillation (AF) or atrial flutter; (ii) prior cardiac surgery for congenital heart disease, cardiomyopathy, valvular disease, or severe myocardial infarction; (iii) post-pacemaker implantation complications, including lead dislocation, pacemaker pocket infection, myocardial perforation, or pacemaker syndrome; (iv) severe liver or kidney disease, hyperthyroidism, malignancy, or other serious illnesses; (v) incomplete clinical data.

### Dual-chamber pacemaker implantation

The patient is positioned supine. After routine skin disinfection and draping of the operative field, 1% lidocaine is administered locally 2 cm below the left clavicle along the clavicular line, followed by a 5 cm incision. The subcutaneous tissue is sequentially dissected down to the pectoralis major fascia, creating a 5 × 5 cm pocket. The axillary vein is punctured, a guide wire is inserted, and its entry into the subclavian vein is confirmed by x-ray.

**Figure 1:**
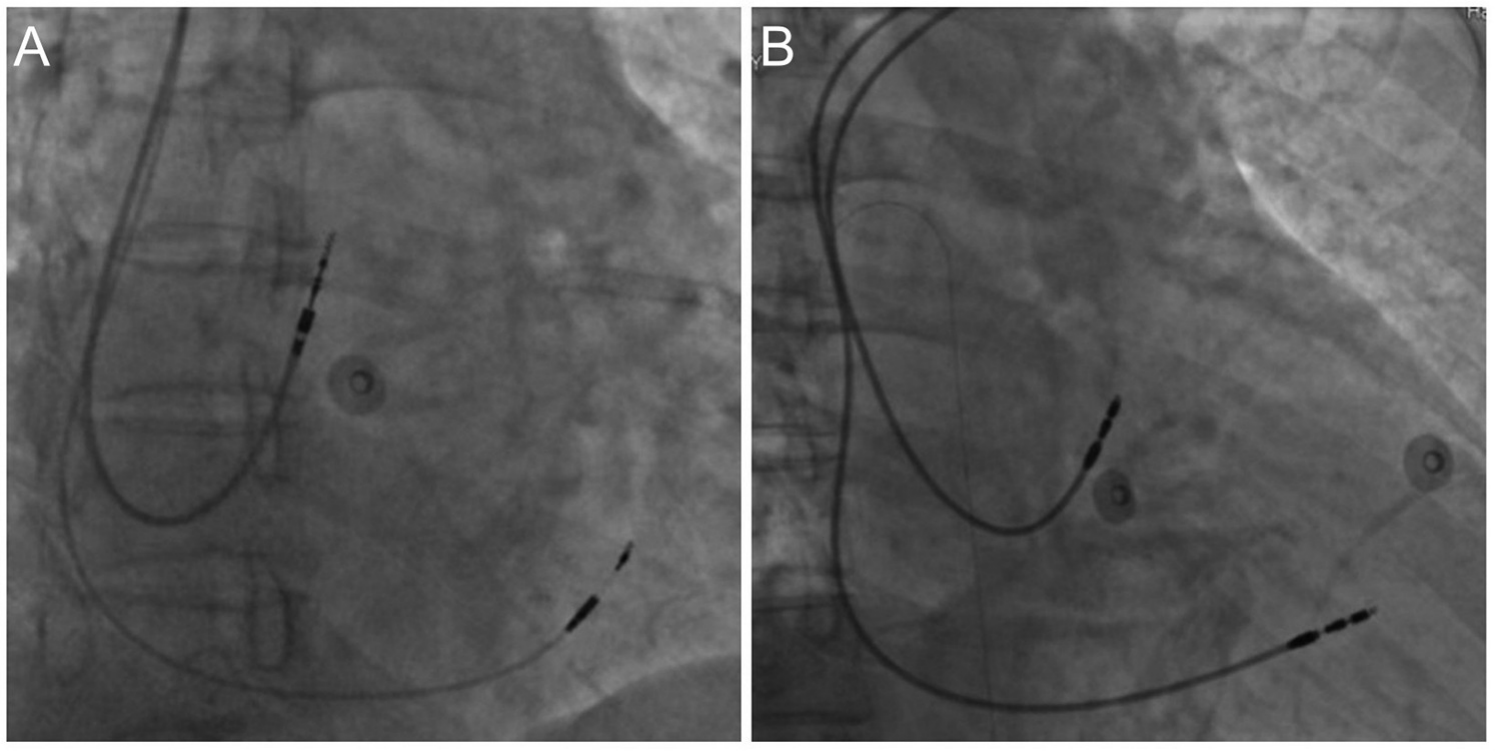
Dual-Chamber pacemaker implantation. **(A)** Left bundle branch pacing; **(B)** right ventricular septal pacing.

For the left bundle branch pacing (LBBP) group, a C315 HIS delivery sheath is introduced along the guide wire. Right ventriculography is performed to display the tricuspid valve. The electrode is then introduced through the sheath, mapping the His bundle. The electrode is advanced and rotated to locate the proximal LBB. Multiple lead physiological recordings are taken to verify the detection of the LBB potential, the pacing electrode tip, and confirm the pacing pattern indicative of right bundle branch block. Additionally, the recordings are used to ensure that the pacing area is specific to LBBP only and not left ventricular septal pacing. Peak time is measured, and parameters such as ventricular sensing, impedance, and bundle branch threshold are tested. If necessary, a dual lead configuration is employed to find the optimal pacing modalities. After successful implantation, the second electrode is retracted to the atrium and positioned at the top of the atrial septum (Figure 1A). In most cases, the right atrial electrode is placed in the right atrial appendage, with only a few cases requiring placement at the atrial septum due to complications.

For the right ventricular septal pacing (RVSP) group, an 8F tear-open sheath is introduced along the guide wire. The right atrium and right ventricular electrodes are introduced through the sheath. The right atrial electrode is secured to the top of the atrial septum, and the right ventricular electrode is fixed to the mid-septum. After electrode placement, testing includes atrial sensing, impedance, and threshold. Pacing the atrium ensures no diaphragmatic contractions. The pulse generator is then connected and placed in the pocket. The electrocardiogram confirms the pacemaker's normal operation (Figure 1B).

The operative area is irrigated with gentamicin and saline, followed by layer-by-layer closure of the pectoralis major and subcutaneous tissue. Tissue glue is used to bond the skin, and the operative site is covered with gauze and dressed for hemostasis.

### Clinical data collection

The collected baseline data include patient demographics such as age and gender, as well as medical histories, including hypertension, coronary heart disease, diabetes, and myocardial infarction. Preoperative laboratory indicators are measured, including serum creatinine, brain natriuretic peptide, cardiac troponin I, total cholesterol, low-density lipoprotein, high-density lipoprotein and triglyceride. Echocardiography parameters such as left ventricular ejection fraction (LVEF), left ventricular end-diastolic diameter (LVEDD), and left atrial diameter (LAD) are also recorded, and the echocardiography is also performed preoperatively. In addition, medication history is documented, as well as the pacemaker lead position.

### Follow up

All pacemaker patients are followed up for at least one year after the procedure. Remote monitoring of pacemaker function is conducted by professional pacemaker engineers using a pacemaker programmer. This includes the pacemaker's working modes, atrial pacing percentage (AP), VP, and documentation of any newly developed diseases. Data regarding new-onset AF are recorded based on electrocardiogram (ECG) results. For patients without AF, data are primarily derived from their examination at the one-year follow-up.

This follow-up approach allows for a comprehensive assessment of patient outcomes, with a particular focus on the development of new-onset AF, which is a key endpoint of the study. The inclusion of both laboratory and echocardiographic parameters helps in understanding the overall cardiac function and the effects of pacemaker therapy in patients with various underlying cardiovascular conditions.

### Statistical analysis

Quantitative data are presented as mean ± standard deviation, while qualitative data are shown as frequency (per centage). Comparisons between groups were performed using the independent two-sample *t*-test. The chi-square test or Fisher's exact test, as appropriate, was used for categorical variables. For the regression analysis, we categorized patients into two groups: none-AF and AF group. This binary classification was used in both univariate and multivariate logistic regression analyses to identify predictors of new-onset AF. A receiver operating characteristic (ROC) curve was generated to determine the optimal cutoffs for indicators with the best diagnostic sensitivity and specificity. Two-sided *P*-values <0.05 were considered statistically significant. All statistical analyses were performed using the IBM SPSS statistical software, version 26.0 (IBM SPSS Inc., Armonk, NY, USA).

## Results

**Figure 2:**
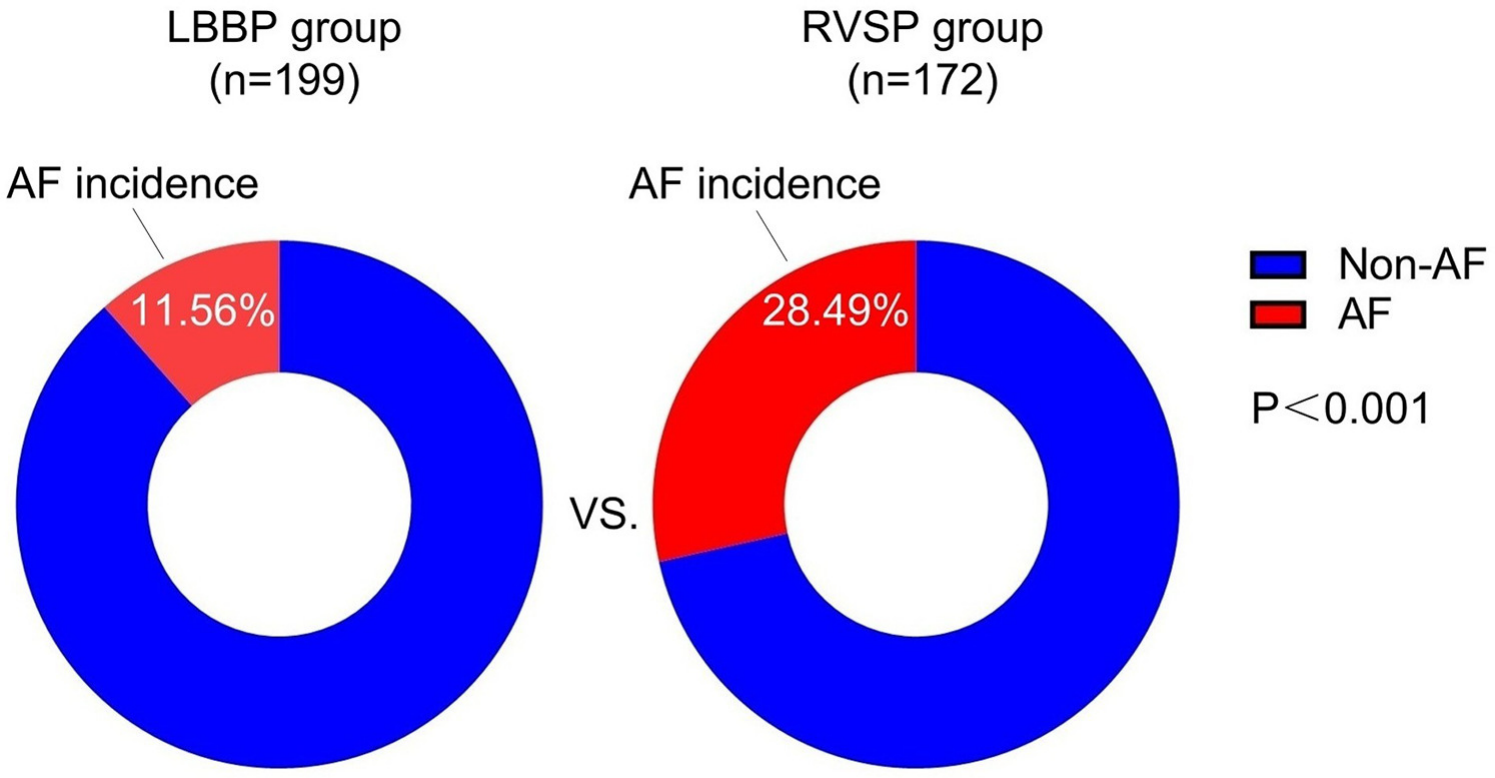
Comparison of the incidence of new-onset AF in relation to different implantation sites of the ventricular lead. AF, atrial fibrillation; LBBP, left bundle branch pacing; RSVP, right ventricular septal pacing.

The baseline characteristics of patients in the non-AF and AF groups are shown in Table 1. The prevalence of hypertension was significantly higher in the AF group compared to the non-AF group (54.17% vs. 37.46%, *P* = 0.010). Regarding the implantation site of the ventricular lead, the proportion of left bundle branch pacing (LBBP) was significantly lower in the AF group compared to the non-AF group (31.94% vs. 58.86%, *P* < 0.001). In terms of ventricular pacing percentage (VP), the proportion of patients with VP 0%–39% was significantly lower in the AF group compared to the non-AF group (23.61% vs. 44.48%, *P* = 0.001). Conversely, the proportion of patients with VP 40%–79% was significantly higher in the AF group compared to the non-AF group (26.39% vs. 11.04%, *P* = 0.002). Figure 2 demonstrates that the incidence of AF was significantly lower in the LBBP cohort compared to the RVSP cohort (11.56% vs. 28.49%, *P* < 0.001).

**Table 1:** Baseline characteristics of patients between non-AF (*n* = 299) and AF group (*n* = 72).

Characteristics	Non-AF group	AF group	*P*-value
Age, years	68.00 ± 3.83	68.50 ± 3.83	0.606
Sex, *n* (%)			
Male	143 (47.83)	37 (51.39)	0.587
Female	156 (52.17)	35 (48.61)	0.587
Primary disease, *n* (%)			0.130
SSS	96 (32.11)	30 (41.67)	
AVB	203 (67.89)	42 (58.33)	
Medical history, *n* (%)			
Hypertension	112 (37.46)	39 (54.17)	**0**.**010**
DM	46 (15.38)	11 (15.28)	0.982
CHD	117 (39.13)	34 (47.22)	0.210
Implantation site of the ventricular lead, *n* (%)			**<0**.**001**
LBBP	176 (58.86)	23 (31.94)	
RVSP	123 (41.14)	49 (68.06)	
AP			0.155
<50%	197 (65.89)	41 (56.94)	
≥50%	102 (34.11)	31 (43.06)	
VP			
0–39%	133 (44.48)	17 (23.61)	**0**.**001**
40–79%	33 (11.04)	19 (26.39)	**0**.**002**
≥80%	133 (44.48)	36 (50.00)	0.430
Medications, *n* (%)			
Beta-blockers	25 (8.36)	7 (9.72)	0.712
Calcium channel blockers	85 (28.43)	19 (26.39)	0.729
Lipid-lowering drugs	119 (39.80)	28 (38.89)	0.887
ACEI/ARB	44 (14.72)	8 (11.11)	0.429

Bold type indicates *P* value < 0.005.

Mean values (standard deviation) and % (n) were reported for continuous and categorical variables, respectively. In the table, the percentages are calculated based on columns. AF, atrial fibrillation; SSS, sick sinus syndrome; AVB, atrioventricular block; DM, diabetes mellitus; CHD, coronary heart disease; LBBP, left bundle branch pacing; RVSP, right ventricular septal pacing; AP, atrial pacing percentage; VP, ventricular pacing percentage; ACEI, angiotensin-converting enzyme inhibitor; ARB, angiotensin receptor blocker.

The characteristics of laboratory testing and echocardiography between the non-AF and AF groups are shown in Table 2. The left atrial diameter (LAD) was significantly larger in the AF group compared to the non-AF group (38.10 ± 1.54 mm vs. 35.90 ± 1.79 mm, *P* = 0.004).

**Table 2:** Characteristics of laboratory testing and echocardiography between non-AF (*n* = 299) and AF group (*n* = 72).

Characteristics	Non-AF group	AF group	*P*-value
cTnI, ug/L			0.842
<0.017	203 (67.89%)	48 (66.67%)	
≥0.017	96 (32.11%)	24 (33.33%)	
Creatinine, μmol/L	79.00 ± 6.88	77.50 ± 5.42	0.632
BNP, pg/ml	269.00 ± 113.60	291.50 ± 100.66	0.880
Total cholesterol, mol/L	4.34 ± 0.94	4.33 ± 0.88	0.977
LDL, mol/L	2.54 ± 0.86	2.55 ± 0.73	0.903
HDL, mol/L	1.15 ± 0.30	1.13 ± 0.31	0.682
Triglyceride, mol/L	1.64 ± 0.75	1.73 ± 0.77	0.453
LAD, mm	35.90 ± 1.79	38.10 ± 1.54	**0**.**004**
LVEDD, mm	46.30 ± 1.58	46.35 ± 1.35	0.504
LVEF, %			0.191
<50%	144 (48.16)	41 (56.94)	
≥50%	155 (51.84)	31 (43.06)	
Mitral Regurgitation			
Mild	79 (26.42)	22 (30.56)	0.465
Moderate	179 (59.87)	41 (56.94)	0.689
Severe	41 (13.71)	9 (12.50)	1.000

Bold type indicates *P* value < 0.005.

Mean values (standard deviation) and % (*n*) were reported for continuous and categorical variables, respectively. In the table, the percentages are calculated based on columns. AF, atrial fibrillation; cTNI, cardiac troponin I; BNP, brain natriuretic peptide; HDL, high-density lipoprotein; LDL, low-density lipoprotein; LAD, left atrial diameter; LVEDD, left ventricular end-diastolic diameter; LVEF, left ventricular ejection fraction.

LBBP [Odds Ratio [OR], 0.315; 95% confidence interval [CI], 0.147–0.600; *P* = 0.011], VP 0%–39% range (OR, 0.613; 95% CI, 0.208–0.913; *P* = 0.024) were independently associated with a reduced risk of new-onset AF, while a larger LAD (OR, 1.017; 95% CI, 1.086–1.228; *P* = 0.025) was independently associated with an increased risk of AF (Table 3). Subgroup analysis for impact of LBBP on new-onset AF in 0%–39% and 40%–79% VP population were shown in Table 4.

**Table 3:** Univariate and multivariate regression analysis for New-onset AF.

Characteristics	Univariate			Multivariate		
	OR	95% CI	*P*-value	OR	95% CI	*P*-value
Age	1.019	0.994–1.183	0.273	–	–	–
Male sex	1.129	1.024–3.034	0.132	–	–	–
Hypertension	4.798	2.162–10.645	0.021	4.646	2.132–10.609	0.121
LBBP	0.264	0.143–0.506	0.001	0.315	0.147–0.600	**0**.**011**
VP 0–39%	0.646	0.214–0.950	0.001	0.613	0.208–0.913	**0**.**024**
VP 40–79%	1.383	1.074–1.839	0.006	1.304	1.067–1.819	0.076
LAD	1.162	1.065–1.266	0.005	1.017	1.086–1.228	**0**.**025**

Bold type indicates *P* value < 0.005.

CI, confidence interval; OR, odds ratio; AF, atrial fibrillation; LBBP, left bundle branch pacing; VP, ventricular pacing percentage; LAD, left atrial diameter.

**Table 4:** Subgroup analysis for impact of LBBP on new-onset AF in 0–39% and 40–79% VP population.

Characteristics	VP 0–39%		VP 40–79%	
	HR (95% CI)	*P*_interaction_	HR (95% CI)	*P*_interaction_
Age, years		0.167		0.092
<65	2.542 (1.199–5.388)		2.206 (1.180–4.126)	
≥65	1.749 (0.630–4.857)		2.055 (1.097–3.850)	
Sex		0.263		0.373
Male	2.051 (0.918–4.587)		1.944 (0.920–4.105)	
Female	2.425 (0.968–6.074)		3.528 (1.047–11.882)	
Hypertension		0.549		0.204
Yes	1.810 (0.837–3.912)		2.212 (1.044–4.684)	
No	2.937 (1.096–7.868)		3.248 (0.978–10.787)	
DM		0.587		0.194
Yes	2.113 (0.918–4.861)		1.751 (0.588–5.211)	
No	2.339 (0.956–5.722)		2.276 (0.955–5.423)	
CHD		0.351		0.265
Yes	1.818 (0.833–3.970)		1.809 (0.937–3.493)	
No	2.812 (1.060 −7.461)		1.043 (0.569–1.909)	
AP		0.637		0.533
<50%	2.261 (1.167–4.378)		1.417 (0.815–2.465)	
≥50%	2.364 (0.511–10.945)		1.506 (0.887–2.558)	
LAD, mm		0.197		0.737
<40	2.237 (1.198–4.175)		1.863 (1.084–3.200)	
≥40	1.500 (0.094–23.981)		2.555 (0.622–10.485)	

CI, confidence interval; HR, hazard ratio; VP, ventricular pacing percentage; AF, atrial fibrillation; LBBP, left bundle branch pacing; LAD, left atrial diameter; DM, diabetes mellitus; CHD, coronary heart disease; AP, atrial pacing percentage.

**Figure 3:**
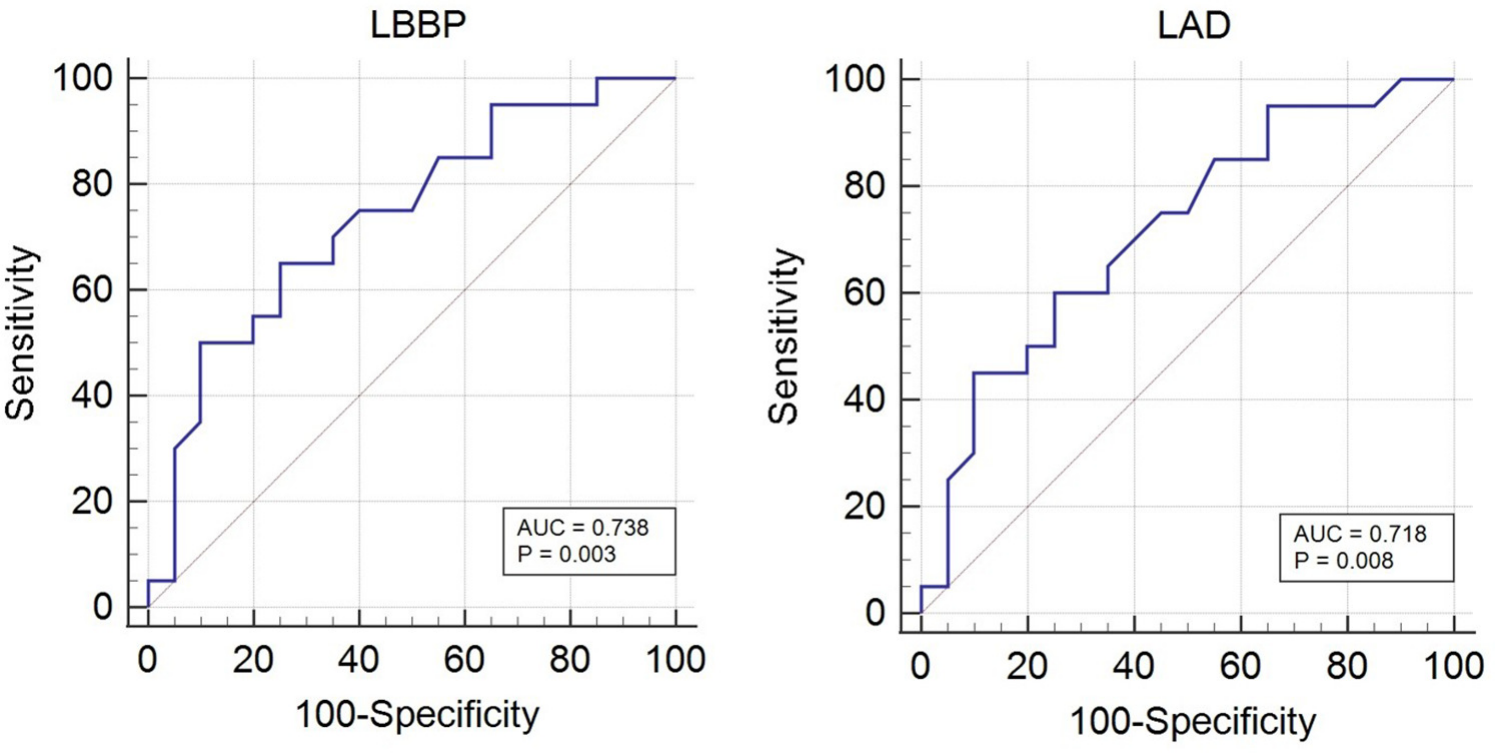
Diagnostic efficiency of LBBP and LAD for new-onset AF using standard ROC curve analysis. AF, atrial fibrillation; LBBP, left bundle branch pacing; LAD, left atrial diameter.

Table 5 and Figure 3 displays the sensitivity and specificity of various indicators in predicting new-onset AF. For LBBP, sensitivity was 65%, specificity was 75%, and AUC was 0.738. For the LAD, sensitivity was 60%, specificity was 75%, and the AUC was 0.718 with the cut-off value of 38.10 mm.

**Table 5:** Diagnostic performance in predicting new-onset AF with different indicators.

Characteristics	LBBP	LAD
AUC	0.738 (0.575–0.863)	0.718 (0.553–0.848)
Cut-off value	–	38.10
Sensitivity	0.650 (0.622–0.678)	0.600 (0.590–0.610)
Specificity	0.750 (0.740–0.760)	0.750 (0.740–0.760)
*P*-value	**0**.**003**	**0**.**008**

Bold type indicates *P* value < 0.005.

AF, atrial fibrillation; LBBP, left bundle branch pacing; LAD, left atrial diameter; AUC, area under the curve.

## Discussion

The principal findings revealed that the AF group had a lower proportion of patients with ventricular pacing (VP) in the 0%–39% range, but a higher proportion of patients in the 40%–79% range. Notably, the incidence of AF was significantly lower in the LBBP group compared to the RVSP group. LBBP, VP in the 0%–39% range, and LAD were independently associated with new-onset AF. These findings underscore the significant influence of both pacing modalities and percentage of ventricular pacing on the risk of new-onset AF following dual-chamber pacemaker implantation. The results suggest that optimizing these factors, particularly by favoring LBBP and minimizing the VP percentage, may play a crucial role in mitigating the risk of AF in patients with pacemakers. This insight provides valuable guidance for clinicians in tailoring pacemaker implantation strategies to potentially reduce the incidence of post-implantation AF.

Pacemaker implantation, while therapeutic, can potentially impact cardiac function through various mechanisms (9). The implantation process itself causes localized myocardial damage due to electrode lead placement. Moreover, the altered cardiac conduction sequence post-implantation can disrupt normal atrial and ventricular contraction patterns, potentially leading to increased atrial pressure (10). These factors, collectively, may contribute to an increased risk of AF (11). Given these considerations, the selection of an optimal pacing modalities is crucial. An ideal pacing location should minimize myocardial damage, preserve physiological conduction patterns as much as possible, and maintain efficient atrial and ventricular function (12). By optimizing the pacing modalities, it may be possible to mitigate the risk of AF associated with pacemaker implantation while ensuring the device's therapeutic efficacy (13). This underscores the importance of personalized approaches in pacemaker therapy, taking into account individual patient characteristics and the potential long-term consequences of pacing on cardiac electrophysiology and mechanics.

LBBP is an advanced technique derived from His bundle pacing (HBP) (14). It involves advancing the pacing electrode through the right ventricular septum and securing it into the endocardial surface of the left ventricle, where it can capture the left bundle branch or its proximal branches. This results in a narrow QRS complex that resembles right bundle branch block. LBBP typically targets myocardial tissue within the interventricular septum under low-output conditions. Compared to right ventricular pacing (RVP), LBBP is associated with a lower incidence of new-onset AF. Research has demonstrated that in patients with a ventricular pacing burden of ≥20%, LBBP significantly reduces the risk of new-onset AF, with the most notable reductions observed for episodes lasting ≥30 s and ≥6 min. Specifically, LBBP has been shown to reduce the relative risk of AF lasting ≥30 s by 67%, yielding an absolute risk reduction of 13% (15). After adjusting for other predisposing factors for AF, some studies suggest that LBBP may act as an independent protective factor against the development of new-onset AF. Patients with a high ventricular pacing burden, particularly those with a VP ≥20%, may experience more pronounced benefits from LBBP, with greater reductions in the risk of AF onset.

Apart from electrode position, the VP is also a critical factor in the development of new-onset AF following pacemaker implantation. Previous studies have suggested an association between minimal ventricular pacing (MVP) and a reduced risk of AF (16). In the SAVE-PACE randomized trial, the MVP group demonstrated significantly lower ventricular pacing rates (9.1% vs. 99.0%) and a reduced incidence of AF (7.9% vs. 12.7%) compared to the dual chamber pacing group (17). The exact mechanisms by which ventricular pacing contributes to AF remain not fully understood, but it may be linked to asynchronous ventricular pacing and interventricular desynchronization, which could lead to abnormal ventricular excitations (18). Electromechanical desynchrony may have long-term detrimental effects on cardiac structure and function (19). Over time, ventricular remodeling can impair both contraction and relaxation, contribute to mitral regurgitation, and increase LAD (20). These changes collectively disrupt left ventricular hemodynamics, thereby promoting the development of AF.

In our future clinical research, we will place particular emphasis on the monitoring of atrial high rate episodes (AHREs), given their increasing significance in pacemaker recipients. AHREs, although not synonymous with clinical AF, have been recognized as important precursors or predictors for the development of AF, especially when these episodes last longer than 5–6 min (21). Studies have shown that AHREs are closely linked to the onset of AF (22) and associated thromboembolic events (23), highlighting the importance of their detection in identifying patients at higher risk. The detection and monitoring of AHREs using implanted devices, such as pacemakers and defibrillators, is critical in providing valuable insight into the risk of AF and its associated complications (23). Optimizing factors such as atrial pacing percentage and minimizing ventricular pacing in pacemaker patients can reduce the risk of new-onset AF, as a higher ventricular pacing burden is associated with an increased risk of AF development.

### Limitation

First, this study was conducted at a single center, which may limit the external validity of the findings. Results may not be applicable to other populations or healthcare settings with different patient demographics or treatment protocols. Second, the retrospective study design carries the risk of inherent biases and limits the ability to establish causality, which could lead to over-correction and unreliable estimates. Third, data on new-onset AF were primarily based on ECG results, which were performed for patients who exhibited symptoms related to AF. However, this approach might not capture all cases, especially in patients with intermittent or asymptomatic AF. Alternative methods such as Holter monitoring or continuous ECG recordings could provide a more accurate assessment. Finally, the outcomes related to different pacing modalities (LBBP vs. RVSP) may be influenced by the experience and technique of the operators performing the implantation, which could introduce variability in the results.

## Conclusions

Ventricular pacing modalities and percentage significantly impact new-onset AF after dual-chamber pacemaker implantation. LBBP and lower VP percentages are associated with a lower risk of AF development, whereas a larger LAD may be linked to an increased likelihood of its onset. Optimizing these factors could potentially reduce the risk of AF in pacemaker patients.

## Data Availability

The raw data supporting the conclusions of this article will be made available by the authors, without undue reservation.
